# Nationwide Study of Pediatric Drug-Resistant Epilepsy in Estonia: Lower Incidence and Insights into Etiology

**DOI:** 10.3390/pediatric18010008

**Published:** 2026-01-06

**Authors:** Stella Lilles, Klari Heidmets, Kaisa Teele Oja, Karit Reinson, Laura Roht, Sander Pajusalu, Monica H Wojcik, Katrin Õunap, Inga Talvik

**Affiliations:** 1 Children’s Clinic, Institute of Clinical Medicine, University of Tartu, 50406 Tartu, Estonia; 2 Children’s Clinic, Tartu University Hospital, European Reference Network EpiCARE, 50406 Tartu, Estonia; 3 Genetics and Personalized Medicine Clinic, Institute of Clinical Medicine, University of Tartu, 50406 Tartu, Estonia; 4 Genetics and Personalized Medicine Clinic, Tartu University Hospital, 50406 Tartu, Estonia; 5 Divisions of Newborn Medicine and Genetics and Genomics, Department of Pediatrics, Boston Children’s Hospital, Harvard Medical School, Boston, MA 02115, USA; 6 Manton Center for Orphan Disease Research, Division of Genetics and Genomics, Department of Pediatrics, Boston Children’s Hospital, Harvard Medical School, Boston, MA 02115, USA; 7 Broad Center for Mendelian Genomics, Broad Institute of MIT and Harvard, Cambridge, MA 02142, USA; 8 Tallinn Children’s Hospital, European Reference Network EpiCARE, 13419 Tallinn, Estonia

**Keywords:** pediatric epilepsy, drug-resistant epilepsy, epidemiology, incidence, etiology, genetics, neuroimaging

## Abstract

Background/Objectives: Drug-resistant epilepsy (DRE) is a significant health problem leading to cognitive impairment and reduced quality of life. This study aimed to investigate the incidence and etiology of DRE in children in Estonia. Methods: A retrospective, population-based study of childhood DRE was conducted in Estonia from 1 January 2013, to 31 December 2017. All cases were identified through the only two pediatric neurology departments in the country, both located at tertiary care hospitals (Tartu University Hospital and Tallinn Children’s Hospital), ensuring complete nationwide coverage. Epidemiological, magnetic resonance imaging (MRI), and genetic data (chromosomal microarray, single-gene tests, gene panels, and exome/genome sequencing) were collected. Results: The incidence rate of childhood epilepsy was 84.1 per 100,000. DRE developed in 10% of children with new-onset epilepsy, corresponding to an incidence rate of 8.5 per 100,000. Etiologically relevant MRI abnormalities were identified in 43% of patients with DRE, most commonly congenital brain malformations (19%). Pathogenic single-gene sequence variants were detected in 25 of 110 patients (23%), copy number variants in four patients (4%), and chromosomal aberrations in one patient (1%). Novel candidate disease genes of uncertain pathogenicity were identified in four patients (4%). The most frequent etiology of DRE was structural (29%), followed by genetic (19%), with combined etiologies (13%) also contributing significantly. Conclusions: Our study is the first epidemiological study of DRE in children in Estonia and the Baltic region. The relatively low incidence observed may reflect the comprehensive national ascertainment and centralized management of pediatric epilepsy in tertiary care centers.

## 1. Introduction

Epilepsy is defined as a chronic brain disorder characterized by a persisting predisposition to generate seizures and by the neurobiological, cognitive, psychological, and social consequences of seizure recurrences [1,2]. Epilepsy is one of the most common neurological disorders worldwide, with a lifetime prevalence of 7.6 per 1000 population [3]. The incidence of childhood epilepsy ranges from 41 to 187 per 100,000 being highest in the first year of life and declining to adult levels by the end of ten years [4].

Drug-resistant epilepsy (DRE) affects one-third of persons with epilepsy, and it is defined by the International League Against Epilepsy (ILAE) as ongoing epileptic seizures despite two tolerated, appropriately chosen, and used antiseizure medication (ASM) regimens [5,6]. DRE is a significant health problem resulting in increased risk of sudden unexplained death in epilepsy, injuries, psychosocial dysfunction, cognitive impairment, and reduced quality of life [7,8].

A systematic review and meta-analysis of DRE shows that the pooled incidence ranges from 15% in children under 17 years of age to 34% in adults [9]. The pooled incidence is 24% across studies that included both children and adults [9]. According to the Scottish epidemiological survey of early childhood epilepsies (under three years of age), 36% of children develop DRE 24 months after presentation [10].

Epilepsy etiology is categorized into six groups: structural, genetic, infectious, metabolic, immune, and unknown. Sometimes, more than one category applies—such as genetic-structural etiology in patients with tuberous sclerosis [11].

Identification of genetic etiology and presentation with seizures before the age of 12 months are determinants of DRE [10]. Abnormal electroencephalography (EEG) and neuroimaging, status epilepticus, symptomatic etiology, intellectual disability (ID), abnormal neurological exam, multiple seizure types, and febrile seizures have also been identified as risk factors for DRE [12,13,14].

We have conducted a retrospective population-based epidemiological study of DRE in children who have been newly diagnosed with epilepsy during a five-year study period in Estonia. This study aims to investigate the incidence and etiology of DRE in children in Estonia.

## 2. Materials and Methods

### 2.1. Patients

A retrospective, population-based epidemiological study of childhood DRE was conducted from 1 January 2013 to 31 December 2017 at the Children’s Clinic of Tartu University Hospital and Tallinn Children’s Hospital in Estonia. Estonia is a small country with a population of approximately 1.3 million during the study period and has only two pediatric neurology departments, located in these two hospitals. According to national clinical practice, epilepsy in children is diagnosed exclusively by pediatric neurologists.

### 2.2. Study Setting and Demographics

In Estonia, the patient population is not ethnically homogeneous, as the majority of the population is of ethnic Estonian descent (about 69%). However, there is a significant Russian-speaking minority (around 25%), particularly in cities like Tallinn (the capital) and Narva. Smaller ethnic groups, such as Ukrainians, Finns, Belarusians, Latvians, and Lithuanians, make up about 6% of the population [15].

Both Tartu University Children’s Clinic and Tallinn Children’s Hospital are tertiary care centers with comprehensive, digitized medical record systems, and nearly all pediatric neurologists in Estonia work in these institutions. Therefore, the study included all children in Estonia with DRE whose epilepsy was first diagnosed during the study period and who met the inclusion criteria.

The inclusion criteria were as follows:

(1) DRE as defined by ILAE—“failure of adequate trials of two tolerated and appropriately chosen and used antiepileptic drug (AED) schedules (whether as monotherapies or in combination) to achieve sustained seizure freedom”. Patients prescribed a third ASM regimen due to the ineffectiveness of prior treatments were selected;
(2) Age ≤18 years;
(3) Permanent residence in Estonia, including children of diverse ethnic backgrounds, ensuring continuous access to healthcare services.

### 2.3. Patient Ascertainment

The diagnosis of epilepsy was confirmed by a pediatric neurologist in accordance with the ILAE operational clinical definition of epilepsy: (1) at least two unprovoked (or reflex) seizures occurring more than 24 h apart; (2) one unprovoked (or reflex) seizure with a probability of further seizures similar to the general recurrence risk (at least 60%) following two unprovoked seizures; and (3) diagnosis of an epilepsy syndrome [2].

Patients were identified by retrieving all children with a first-time diagnosis of epilepsy between 1 January 2013 and 31 December 2017 from the electronic databases of the Children’s Clinic of Tartu University Hospital and Tallinn Children’s Hospital. The International Classification of Diseases, 10th Revision (ICD-10) codes G40 and G41 were used to search both outpatient and inpatient records for initial epilepsy diagnoses.

Following case identification, electronic medical records of all patients were reviewed in detail from the time of epilepsy diagnosis (2013–2017) through 1 September 2020, to ensure that (1) the recorded year of first epilepsy diagnosis fell within the study period, and (2) the epilepsy diagnosis was confirmed, not merely suspected and later disproved.

Based on this review, patients were included in the present study if they met criteria for DRE, defined as failure to achieve seizure control with two appropriately chosen and tolerated ASM regimens, with a third ASM initiated after the prior two were ineffective.

### 2.4. Clinical Information and Results of Etiological Investigations

Clinical data of patients with DRE were collected through a comprehensive review of medical records. The following information was obtained: full medical history (including birth and family history), clinical examination findings, seizure characteristics (age at seizure onset, seizure types at onset and during the disease course, and seizure frequency at the time the third ASM regimen was initiated), age at epilepsy diagnosis, epilepsy syndrome classification, and treatment history (types and combinations of ASMs, duration of treatment trials, use of glucocorticoids or ketogenic diet, and surgical interventions).

Results of repeated investigations during the course of epilepsy were also extracted, including cognitive assessments, EEG, and magnetic resonance imaging (MRI) of the brain. Genetic testing results—including chromosomal microarray analysis, next-generation sequencing (NGS) techniques such as single-gene sequencing, targeted gene panels, whole-exome sequencing (WES), and/or whole-genome sequencing (WGS), and, in some cases, karyotyping)—were collected from clinical diagnostic evaluations or research studies, provided that results were validated in a certified diagnostic laboratory.

The selection of a particular genetic test was primarily determined by clinical characteristics and medical indications, rather than cost or availability. The decision to perform a specific test was guided by the patient’s clinical presentation, including factors such as the type of epilepsy, age at onset, family history, and the suspected underlying etiology of the seizures. For example, WES or WGS were considered when more complex or undiagnosed cases were present, or when previous genetic testing failed to identify a cause.

Most genetic testing (including chromosomal microarray analysis, single-gene sequencing, targeted gene panels, WES, and, in some cases, karyotyping) was performed in the in-house laboratory of the Genetics and Personalized Medicine Clinic at Tartu University Hospital. These tests were covered by Estonian national healthcare insurance, which is funded by the Estonian government. A few single-gene sequencing and targeted gene panels were initially performed in commercial laboratories abroad before such testing became available in Estonia. However, these tests were still covered by Estonian national healthcare insurance through an individual reimbursement request. WGS was conducted in research setting at the Broad Institute, supported by grants from the Estonian Research Council.

As a result, the cost of genetic testing was not directly covered by the patients. Financial considerations did not influence the selection of tests, as decisions were made based on clinical necessity rather than cost, and all tests were performed in accordance with established medical protocols.

### 2.5. Statistical Methods

Incidence rates of epilepsy and DRE were calculated as the number of newly diagnosed cases per population at risk, expressed per 100,000 person-years. Population denominators were obtained from Statistics Estonia (www.stat.ee (accessed on 4 January 2024)) and included individuals aged 18 years or younger at the beginning of each year. The mean population of children and adolescents in Estonia between 2013 and 2017 was 257,996 per year, remaining stable during the study period (range 256,249–261,253). A cumulative denominator of 1,289,979 was used to estimate the total population aged <19 years over the five-year period. Age- and sex-specific incidence rates of DRE were calculated similarly. Ninety-five percent confidence intervals (95% CIs) were estimated using the Poisson distribution.

## 3. Results

### 3.1. Incidence

According to hospital medical records, epilepsy was first diagnosed between 2013 and 2017 in 681 patients at the Children’s Clinic of Tartu University Hospital and in 761 patients at Tallinn Children’s Hospital. After a detailed review of all 1442 (*n* = 681 + 761) epilepsy-related records from both hospitals, patients were excluded from the final analysis if they had been diagnosed before the study period, were not Estonian permanent residents, or had a suspected diagnosis that was not later confirmed.

Following this exclusion process, 573 patients at Tartu University Hospital and 554 patients at Tallinn Children’s Hospital remained with a first-time epilepsy diagnosis. Forty-two of the 1127 patients (including 43% with DRE) were treated at both hospitals. Thus, the total number of children in Estonia with a first-time epilepsy diagnosis from 2013 to 2017 was 1085.

The overall incidence of childhood epilepsy was 84.1 per 100,000 children (95% CI 79.2–89.3). Of these, 110 children (10%) had DRE, defined as failure to achieve seizure control with two appropriately chosen and tolerated ASM regimens, with a third regimen subsequently initiated. Table 1 presents the numbers and percentages of newly diagnosed epilepsy and DRE patients by year of diagnosis. On average, 217 children were newly diagnosed with epilepsy and 22 with DRE per year in Estonia.

Fifty-seven of 110 patients with DRE (52%) were male, and 53 (48%) were female, resulting in a male-to-female ratio of 1.1:1. The mean age at epilepsy diagnosis was 4.7 years (95% CI 4.0–5.5), with males averaging 4.7 years (95% CI 3.7–5.6) and females 4.8 years (95% CI 3.6–6.0).

The overall incidence of DRE in children was 8.5 per 100,000 person-years, with similar rates in males (8.6 per 100,000) and females (8.5 per 100,000). The highest incidence (17.7 per 100,000) was observed in children under four years of age, whereas the lowest incidence (0.8 per 100,000) occurred in adolescents aged 15–18 years. In children aged 5–9 and 10–14 years, the incidence rates were 8.9 and 3.5 per 100,000 person-years, respectively. Table 2 presents the age- and sex-adjusted incidence rates of DRE per 100,000 person-years.

### 3.2. Neuroimaging

Brain MRI was performed in 108 of 110 patients with DRE (98%). Depending on availability, 3 Tesla MRI was performed in 47 patients (44%) and 1.5 Tesla MRI in 61 patients (56%). Neuroimaging was not performed in two patients with idiopathic generalized epilepsy (childhood and juvenile absence epilepsy). As these patients were followed and treated within routine clinical practice rather than a research context, neuroimaging is not indicated in idiopathic generalized epilepsy with typical clinical and EEG features, and was therefore not expected to alter diagnosis or management.

Among the 108 patients with DRE, MRI was completely normal in 40 (37%) and showed no clinically relevant pathology in 16 (15%), for a total of 56 patients (52%). These nonspecific, non-relevant findings included small subcortical white matter lesions, hippocampal malrotation, small benign cysts, subtle ventricular widening, and benign enlargement of the subarachnoid space in infancy. In addition, six patients exhibited either brain atrophy or progression of atrophy on consecutive MRIs without other structural pathology; four of these six patients (67%) had additional genetic abnormalities that may have contributed to the development of atrophy.

Etiologically relevant MRI changes were observed in 46 of 108 patients (43%). The most common structural etiology was congenital brain malformation, identified in 20 patients (19%), of whom eight (40%) had focal cortical dysplasia. Other structural causes included perinatal stroke (*n* = 4) and white matter injury or intraventricular hemorrhage associated with preterm birth (*n* = 4). Inflammatory changes were observed in four patients with autoimmune encephalitis and in one patient with acute disseminated encephalomyelitis (ADEM). Three patients each had hypoxic–ischemic perinatal injury, brain tumor, or traumatic brain injury. Tuberous sclerosis was identified in two patients, mesial temporal sclerosis in one patient, and a structural change associated with Herpes Simplex Virus Type 2 (HSV2) encephalitis in one patient (Table 3).

### 3.3. Genetic Testing

Genetic testing included chromosomal microarray analysis, NGS techniques such as single-gene sequencing, targeted gene panels, WES, and/or WGS, and, in some cases, karyotyping. Ninety-two of 110 patients with DRE (84%) underwent at least one genetic analysis. Chromosomal microarray analysis was performed in 69 patients (63%), targeted gene panels in 78 patients (71%), and single-gene sequencing in four patients (4%). WES and/or WGS was conducted in 21 patients (19%).

Table 4 presents the extent of genetic analyses performed in each patient, as determined by the managing child neurologist and clinical geneticist. The largest group of patients (*n* = 42, 38%) received both chromosomal microarray analysis and a gene panel. Chromosomal microarray, gene panel, and WES/WGS were performed in 14 patients (13%). Genetic testing was not performed in 18 patients, most likely due to the absence of a standardized protocol, as these patients were managed within routine clinical practice rather than a research setting. This group included ten patients with a known structural and/or immune etiology for their DRE, for whom genetic testing was not routinely offered. Genetic testing was also not performed in eight patients with unknown etiology, likely due to the absence of a standardized protocol. These patients had no global developmental delay (GDD) or ID, and therefore genetic testing was not included in their routine management.

Of the total cohort of 110 patients with DRE, genetic testing was not performed in 18 patients (16%). Among the 92 patients who underwent genetic testing, 48 (52%) had normal findings. Genetic variants were identified in 39 patients. These included pathogenic sequence variants classified according to the American College of Medical Genetics and Genomics (ACMG) criteria in 25 patients (23% of the total cohort) and variants of uncertain significance (VUS) in 10 patients (9%). Reanalysis of sequencing data revealed novel candidate disease genes in four patients (4%); however, the pathogenicity of these variants remains uncertain.

Copy number variants (CNVs), which are detectable by microarray analysis, were identified in four patients (4%), including one case each with a 10q26.3 deletion, a 15q13.3 microdeletion, a 22q11.2 deletion, and a 22q11.2 microduplication. In addition, one chromosomal aberration—ring chromosome 14—was identified (1%). Table 5 summarizes the pathogenic genetic findings. Two of the pathogenic findings identified in this cohort had been previously reported [16,17]. Detailed sequence variant findings, including pathogenic, VUS, and novel candidate variants, are provided in Table 6. All sequence variants, including nucleotide and protein changes, as well as chromosomal aberrations, are listed in Supplementary Table S1.

Table 6 shows that the most common pathogenic epilepsy-related gene variants were in *MECP2*, *PCDH19*, *SCN1A*, and *TSC2*, each detected in two patients with DRE. Other pathogenic variants in genes were each found in a single patient, including *CDKL5* [16], *COL4A1*, *CPA6*, *CSNK2A1*, *DNM1*, *DYNC1H1*, *GABRG2*, *IRF2BPL*, *KCNQ2*, *KMT2D*, *LAMB1*, *PPT1*, *PRRT2*, *SLC2A1*, *SMARCB1*, *SYNGAP1*, and *UNC13D.* The latter gene was found to harbor biallelic pathogenic variants in one patient with primary hemophagocytic lymphohistiocytosis who subsequently developed ADEM.

Among the novel disease gene candidates, functional studies are ongoing to assess the pathogenicity of biallelic *SIRT6* variants identified in siblings within a single family. One patient had biallelic variants in *ACSL5*, a novel candidate gene; however, these variants did not fully explain the patient’s phenotype. Recently, a second *de novo* variant was detected in the *RNU2-2P* gene (corresponding to the *WDR74* 5’ UTR region), which has been reported as pathogenic [18]. In one patient, two different novel candidate genes—*LMTK3* and *DSCAM*—were identified, with *DSCAM* prioritized as the more plausible candidate.

At present, the pathogenicity of these novel candidate genes remains uncertain, and further functional studies are required to clarify their role in epilepsy.

### 3.4. Comorbidities and Etiology

In our cohort of patients with DRE, the prevalence of ID or significant GDD is 53% (*n* = 58). Additionally, 24 patients (22%) have isolated speech or language delay without ID. Nine patients (8%) have a diagnosis of attention-deficit/hyperactivity disorder (ADHD), six patients (5%) have autism spectrum disorder (ASD), and three patients (3%) have been diagnosed with both ADHD and ASD.

The etiology of epilepsy was identified in 67 of 110 patients (61%) whose epilepsy was first diagnosed between 2013 and 2017 (Table 7). The most frequent causes were structural and genetic, occurring either alone or in combination with other etiologic factors. However, in 39% of patients, the etiology remained unknown.

Among the 43 DRE patients with an unknown etiology, 31 (72%) did not have ID. In this group, nearly half of the patients were diagnosed with self-limited epilepsy with centrotemporal spikes (*n* = 14, 45%). Focal epilepsy without a specific epilepsy syndrome was identified in five patients (16%), and idiopathic generalized epilepsy, including two cases of childhood absence epilepsy and one case of juvenile absence epilepsy, was present in eight patients (26%). Epilepsy with myoclonic atonic seizures was observed in two patients (6%), and epilepsy with myoclonic absences in one patient. Additionally, one child was diagnosed with infantile epileptic spasms syndrome, which required three ASMs to control the seizures. Fortunately, after this treatment, the child became seizure-free and did not develop GDD.

In contrast, 12 (28%) presented with both DRE and ID, suggesting the possibility of an undetected underlying cause. Most of these 12 patients were diagnosed with one of the following syndromes: infantile epileptic spasms syndrome, epilepsy with myoclonic absences, developmental and/or epileptic encephalopathy with spike-wave activation in sleep or epilepsy with myoclonic atonic seizures. Based on these findings, we refined the unknown-etiology category within the full cohort (43 of 110 patients; 39%) into two subgroups: unknown etiology without ID or GDD (*n* = 31; 28%) and unknown etiology with ID or GDD (*n* = 12; 11%).

A single etiological factor for DRE was identified in nearly half of the patients: a solely structural cause in 32 patients (29%) and a pathogenic genetic cause in 21 patients (19%) (Table 8). Among strictly structural causes, the most common were congenital brain malformations, present in 15 patients (14%). White matter injury or intraventricular hemorrhage associated with preterm birth was identified in four patients. Three patients each had perinatal stroke, brain tumor, hypoxic–ischemic perinatal injury, or traumatic brain injury.

A solely genetic cause was identified in 21 of 110 patients (19%). Among these, 16 children (15%) had pathogenic single-gene variants, most frequently in *MECP2*, *SCN1A*, and *PCDH19*, each found in two different patients. DRE in four patients was associated with CNVs, and in one patient with a ring chromosome 14.

We found that combined etiology was also an important factor in children with DRE, identified in 14 of 110 patients (13%) (Table 9). The most frequent combination was genetic–structural etiology, observed in 7 patients (6%). Among these, four patients had pathogenic single-gene variants along with congenital brain malformations. Two patients had tuberous sclerosis caused by pathogenic variants in the *TSC2* gene, and one patient carried a *COL4A1* variant predisposing to perinatal hemorrhagic stroke.

The second most common combined etiology was immune–structural, observed in five patients (5%), including four with autoimmune encephalitis and one with ADEM.

## 4. Discussion

According to our study, the incidence rate of epilepsy in Estonia among the patients under 19 years of age is 84.1 per 100,000 persons-years (95% CI 79.2–89.3). This finding is consistent with a previous Estonian study conducted between 2009 and 2011, which reported an incidence rate of 86.3 per 100,000 person-years [19]. In contrast, a childhood epilepsy study conducted in Estonia from 1995 to 1997 reported a lower overall incidence of 45 per 100,000 children aged 1 month to 19 years, with the highest rate observed in children under four years of age (75 per 100,000) [20]. The increase in incidence over time likely reflects improved diagnostic methods, better access to pediatric neurologists, the availability of EEG in tertiary care centers, and digitized medical records.

Comparisons with other epidemiological studies should be made cautiously due to differences in study methodologies [21]. The incidence rate observed in our study is broadly comparable to reports from Nordic countries, which range from 25 to 87 per 100,000 person-years (Finland: 25–87; Sweden: 40–82; Norway: 47) [21,22,23,24,25,26,27,28,29]. Another Norwegian study reported an incidence of 58 per 100,000 person-years in children aged 1–10 years and 144 per 100,000 in the first year of life [30]. The comparison with Nordic countries was made based on methodological and healthcare system similarities. Both Estonia and the Nordic countries have well-organized, publicly funded healthcare systems and centralized patient registries, which allow for more reliable and consistent data collection, making these countries suitable for comparative epidemiological research [31].

Unlike previous Estonian childhood epilepsy incidence studies, which examined two earlier periods, our study specifically focused on the development of DRE in newly diagnosed epilepsy patients. To our knowledge, this is the first epidemiological study of DRE in children in the Baltic countries. We found that the incidence of DRE in Estonian patients under 19 years of age is 10%, which is lower than reported in most literature. A recent systematic review and meta-analysis reported a cumulative DRE incidence of 25% in pediatric studies and 14.6% in adult or mixed-age populations [32]. Our estimate aligns more closely with other meta-analyses reporting a pooled pediatric incidence of 15% (<17 years) [9].

Current guidelines suggest that patients who fail to achieve seizure control with two appropriately chosen and tolerated ASM regimens should be referred to a comprehensive epilepsy center [33]. In Estonia, there are only two pediatric neurology departments, both located at tertiary care centers, where all patients with new-onset epilepsy are treated. We hypothesize that early management in high-expertise tertiary centers may reduce the risk of progression to DRE, potentially explaining the lower incidence observed in our study. Further research is needed to evaluate whether early referral to centers of expertise improves treatment responsiveness and long-term prognosis.

Differences in DRE definitions across studies also affect incidence estimates. Among 38 studies on pediatric DRE, only four (10.5%) fully adhered to the ILAE criteria regarding the number of failed ASMs, appropriateness of ASM treatment, and the period for assessing seizure remission [9]. Although most prior studies predate the 2010 ILAE guidelines, we applied strict DRE criteria in our study, including only patients who initiated a third ASM regimen. It is possible that some patients whose seizures were not controlled by the second ASM were not captured because a third regimen was not prescribed, which may further explain the lower incidence observed in our cohort.

Our study provides overall and age- and sex-specific incidence rates of DRE per 100,000 person-years, which have not previously been reported in the literature. The overall incidence of DRE in patients under 19 years was 8.5 per 100,000 person-years (95% CI 7.0–10.3). Symonds et al. reported a DRE incidence of 82 per 100,000 live births in Scotland among patients whose epilepsy was diagnosed before the age of three years [10].

In our cohort, the incidence was similar between genders, with 8.6 per 100,000 for males and 8.5 per 100,000 for females, consistent with other studies showing no significant sex differences [10,34]. The highest incidence was observed in children whose epilepsy was diagnosed under four years of age, particularly in infants under one year (36.1 per 100,000). This finding aligns with previous reports indicating that the highest incidence of epilepsy occurs in the first years of life [35] and that seizure onset during the first year of life is a recognized risk factor for drug resistance [36].

Our study focused on the development of DRE in newly diagnosed, rather than chronic, epilepsy patients; therefore, prevalence estimates are not provided. Reported prevalence of DRE varies from 13.7% in population- or community-based cohorts to 36.3% in clinic-based cohorts [28].

Brain MRI was performed in the majority of patients (98%), with etiologically relevant abnormalities observed in 43% of children with DRE. Previous studies have shown that the risk of developing DRE is nearly three times higher in patients with symptomatic compared to idiopathic epilepsy [9] and abnormal neuroimaging is consistently associated with an increased risk of drug resistance [9,14,37].

In our cohort, structural abnormalities were less frequent than reported in other studies, which ranged from 76% to 80.7% [14,37]. The most common structural abnormality was congenital brain malformations, including focal cortical dysplasia, present in 19% of patients. This finding is similar to that reported by Nasiri et al., who identified cerebral dysgenesis in 17.6% of patients with intractable seizures [37]. Less frequent structural findings included perinatal stroke, white matter injury or intraventricular hemorrhage associated with preterm birth, inflammatory changes related to autoimmune encephalitis or ADEM, hypoxic–ischemic perinatal injury, brain tumors, and traumatic brain injury.

Pathogenic sequence variants were identified in 23% of patients with DRE. Additionally, 4% of patients harbored candidate disease-causing variants; however, their pathogenicity remains uncertain, and they were therefore not considered contributory to the genetic etiology in our cohort. Copy number variants were detected in 4% of patients, and chromosomal aberrations in 1%. Compared with previous reports, the proportion of pathogenic sequence variants in our cohort was somewhat lower than the 30–40% reported for developmental and epileptic encephalopathies, whereas the frequency of chromosomal deletions and duplications remained within the 5–10% range previously described [38,39]. The lower proportion of pathogenic sequence variants in our cohort may reflect differences in cohort composition, inclusion criteria, or the scope of genetic testing, given that 16% of patients were not tested.

There have been significant advances in the discovery of disease-related genes, and to date, more than 900 genes are associated with epilepsy [40,41]. According to the meta-analysis of genetic causes of DRE and the study by Lin et al., the most common gene variants have been identified in *SCN1A*, *PCDH19*, *SCN8A*, *SCN2A*, *MECP2*, *KCNQ2*, *CDKL5*, *TSC1*, and *TSC2*, which are also found in our patients, except *TSC1* [42]. We identified pathogenic variants in the following genes: *MECP2*, *PCDH19*, *SCN1A*, and *TSC2* in two patients each.

The novel disease gene candidate *SIRT6* is less well characterized than the previously mentioned genes. *SIRT6* is a histone deacetylase that plays a role in genomic stability, DNA repair, cancer, metabolism, and aging. It has been shown that *SIRT6* participates in brain development, regulating the proliferation of neural precursor cells and cortical neurogenesis [43]. Ferrer et al. reported severe neurodevelopmental and cardiac anomalies and perinatal lethality in four affected fetuses with a homozygous inactivating variant in the *SIRT6* gene [44].

In our study, two triplet siblings carried compound heterozygous variants in *SIRT6*, presented with epilepsy and brain malformations, and two met criteria for DRE. *SIRT6* deficiency is associated with high lethality; all three siblings died within the first two years of life. Functional analyses of *SIRT6* are currently ongoing. Therefore, we classified the etiology of epilepsy in these patients as structural rather than genetic-structural.

Identifying the underlying etiology of DRE is crucial for improving diagnosis, management, and prognosis. In our study, the etiology of DRE was established in 61% of patients, which is lower than previously reported by Nasiri et al. (80.6%) and Symonds et al. (82%) [10,37]. This discrepancy may reflect differences in study design and the fact that genetic analyses were not performed in 8 of 43 patients (19%) with unknown etiology. However, these eight patients did not have GDD or ID. Although etiology was not identified in 39% of patients, it is important to note that many of these patients (28%) did not have ID accompanying their DRE. Conversely, 11% of patients with both DRE and ID/GDD still had an unknown etiology, raising the suspicion of an undetected underlying cause in this group.

We classified the etiology as unknown in five patients whose MRIs were normal or showed no relevant pathology, and who carried variants of unknown significance or candidate disease-causing variants. Further functional studies are required to clarify the potential genetic contribution in these cases.

In our cohort, the most common etiology of DRE was structural (29%), followed by genetic (19%). Combined etiologies were also significant, identified in 13% of patients. The most frequent combined etiologies were genetic-structural (6%) and immune-structural (5%), followed by infectious-structural and genetic-metabolic-structural, each observed in 1% of patients.

These findings are broadly comparable to a Scottish study of children under three years of age with epilepsy, in which 139 children (36%) developed DRE within 24 months of presentation. Structural etiologies were reported in 26.6% of patients, structural-metabolic in 0.7%, and structural-infectious in 1.4%. Notably, the prevalence of immune-structural etiology in our cohort (5%) was higher than in the Scottish study (0.7%), which may reflect that all of our patients with immune-structural etiology were older than three years at epilepsy onset, whereas the Scottish cohort included younger children. Conversely, the frequency of defined genetic etiology was lower in our study than in the Scottish cohort (19% vs. 52.5%) [10].

Overall, the etiology of DRE remained unknown in 39% of our patients, a rate higher than that reported in the Scottish study [10]. Performing genetic analyses in the remaining 19% of patients with unknown etiology who have not yet been tested may reveal additional genetic contributions to DRE in our cohort.

A limitation of our study is the relatively small cohort, which reflects Estonia’s overall small population. Nevertheless, the country’s well-digitized, nationwide medical system enables population-based epidemiological research. Although epilepsy in children in Estonia is expected to be diagnosed by pediatric neurologists, who predominantly work in the only two tertiary pediatric neurology centers, it is possible that a few newly diagnosed cases were missed. We believe, however, that nearly all children with DRE were captured in our study, given the small population, accessible tertiary care centers, and parental healthcare-seeking behavior in cases of more severe epilepsy.

A major strength of our study is the extensive diagnostic evaluation performed in patients with DRE. By providing detailed etiological data, our findings may inform better management of pediatric epilepsy. Our study adds to the literature the hypothesis that treating all newly diagnosed epilepsy patients in tertiary care centers reduces the possibility of drug resistance. However, further exploration of this point will be needed.

## 5. Conclusions

This study represents the first epidemiological investigation of DRE in children in Estonia and the Baltic region. The overall incidence of childhood epilepsy was comparable to studies from Nordic countries and a previous Estonian survey. DRE was identified in 10% of patients, which is lower than commonly reported in the literature, likely reflecting the comprehensive nationwide management of pediatric epilepsy in Estonia’s centralized tertiary care system. The most frequent etiologies were congenital brain malformations and pathogenic gene variants, with combined genetic, immunologic, and structural factors also contributing to DRE development. This unique healthcare setting enabled detailed characterization of the etiological spectrum of DRE.

## Figures and Tables

**Table 1:** Newly diagnosed epilepsy patients and DRE cases (*n*, %) by year of diagnosis.

Year	New Epilepsy Patients (*n*)	Patients with DRE, *n* (%)
2013	216	25 (12%)
2014	220	25 (11%)
2015	204	26 (13%)
2016	226	15 (7%)
2017	219	19 (9%)
Average per year	217	22 (10%)

**Table 2:** Age- and sex-specific incidence rate (per 100,000 children) of DRE.

Age (Years)	Total			Males			Females		
	Persons at risk	Cases (*n*)	Rate (95% CI)	Persons at risk	Cases (*n*)	Rate (95% CI)	Persons at risk	Cases (*n*)	Rate (95% CI)
0–4	362,308	64	17.7 (13.6–22.6)	185,965	33	17.8 (12.2–24.9)	176,343	31	17.6 (11.9–25.0)
5–9	371,781	33	8.9 (6.1–12.5)	191,173	19	9.9 (6.0–15.5)	180,608	14	7.8 (4.2–13.0)
10–14	315,408	11	3.5 (1.7–6.2)	161,948	5	3.1 (1.0–7.2)	153,460	6	3.9 (1.4–8.5)
15–18	240,482	2	0.8 (0.1–3.0)	123,791	0	0 (–)	116,691	2	1.7 (0.2–6.2)
Total	1,289,979	110	8.5 (7.0–10.3)	662,877	57	8.6 (6.5–11.1)	627,102	53	8.5 (6.3–11.1)

**Table 3:** Neuroimaging findings in 46 patients with structurally relevant pathology.

Structural Pathology	Patients (*n*)
Congenital brain malformations (including focal cortical dysplasia)	20 (8)
Perinatal stroke	4
Preterm-related white matter injury or intraventricular hemorrhage	4
Inflammatory changes associated with autoimmune encephalitis	4
Hypoxic–ischemic perinatal injury	3
Inflicted traumatic brain injury	3
Brain tumor	3
Tuberous sclerosis	2
Mesial temporal sclerosis	1
ADEM	1
Structural changes associated with HSV2-encephalitis	1

**Table 4:** Extent of genetic analysis in genetically tested patients with DRE.

Genetic Assay	Patients (*n*)
Chromosomal microarray only	5
Chromosomal microarray and single gene testing	4
Chromosomal microarray and gene panel	42
Chromosomal microarray and WES/WGS	4
Chromosomal microarray and gene panel and WES/WGS	14
Gene panels only	20
Gene panel and WES/WGS	2
WES/WGS only	1

**Table 5:** Pathogenic genetic findings in 30 of 110 DRE patients.

Genetic Finding	Patients, *n* (%)
Pathogenic sequence variants	25 (23%)
CNVs	4 (4%)
Chromosomal aberration	1 (1%)

**Table 6:** Single gene sequence variants (according to ACMG criteria).

Sequence Variants	Patients (*n*)	Genes
Pathogenic	25	Two cases: *MECP2*, *PCDH19*, *SCN1A*, *TSC2* One patient: *CDKL5* [16], *COL4A1*, *CPA6*, *CSNK2A1*, *DNM1*, *DYNC1H1*, *GABRG2*, *IRF2BPL*, *KCNQ2*, *KMT2D*, *LAMB1*, *PPT1*, *PRRT2*, *SLC2A1*, *SMARCB1*, *SYNGAP1*, *UNC13D*
Novel disease gene candidates (unclear pathogenicity)	4	Two cases: *SIRT6* One patient: *ACSL5/RNU2-2P*, *DSCAM/LMTK3*
VUS (nonpathogenic)	10	Two cases: *SCN2A* One patient: *ALG13* [17], *CACNA1E*, *HCN1*, *KIF1A*, *RYR2*, *SLC9A6*, *SPTAN*, *WNK3*

**Table 7:** Etiology of DRE in 110 pediatric patients in Estonia.

Etiology	Patients, *n* (%)
Unknown without ID/GDD	31 (28%)
Unknown with ID/GDD	12 (11%)
Structural (only)	32 (29%)
Genetic (only)	21 (19%)
Genetic-structural	7 (6%)
Immune-structural	5 (5%)
Infectious-structural	1 (1%)
Genetic-metabolic-structural	1 (1%)

**Table 8:** Strictly structural or genetic etiology in DRE patients.

Etiology	Patients, *n (%)*
Structural:	32 (29%)
Congenital brain malformations (including focal cortical dysplasia)	15 (6)
Preterm-related white matter injury or intraventricular hemorrhage	4
Perinatal stroke	3
Brain tumor	3
Hypoxic–ischemic perinatal injury	3
Inflicted traumatic brain injury	3
Mesial Temporal Sclerosis	1
Genetic:	21 (19%)
Pathogenic gene sequence variants: Two cases: *MECP2*, *PCDH19*, *SCN1A* One patient each: *CDKL5* [16], *CPA6*, *DNM1*, *DYNC1H1*, *GABRG2*, *IRF2BPL*, *KCNQ2*, *PPT1*, *PRRT2*, *SYNGAP1*	16
Copy number variants: 10q26.3 deletion, 15q13.3 microdeletion, 22q11.2 deletion, 22q11.2 microduplication	4
Chromosomal aberration: Ring chromosome 14	1

**Table 9:** Combined etiology in DRE patients.

Combined Etiology	Patients, *n* (%)
Genetic-structural:	7 (6%)
*SMARCB1*, *KMT2D*, *LAMB*, *CSNK2A1* pathogenic variants and congenital brain malformations	4
*TSC2* pathogenic variants and tuberous sclerosis	2
*COL4A1* pathogenic variant and perinatal hemorrhagic stroke	1
Immune-structural:	5 (5%)
Autoimmune encephalitis	4
ADEM	1
Infectious-structural:	1 (1%)
HSV2-encephalitis with subsequent porencephaly	1
Genetic-metabolic-structural:	1 (1%)
*SLC2A1* pathogenic variant (GLUT1-DS) and focal cortical dysplasia	1

## Data Availability

Due to privacy and ethical restrictions, the research data are not publicly available; however, all sequence variants, including nucleotide and protein changes, and chromosomal aberrations, are provided in Supplementary Table S1.

## Supplementary Materials

The following supporting information can be downloaded at: https://www.mdpi.com/article/10.3390/pediatric18010008/s1, Table S1: Sequence variants and chromosomal aberrations.

## Abbreviations

The following abbreviations are used in this manuscript:

ACMG	American College of Medical Genetics and Genomics
ADEM	Acute disseminated encephalomyelitis
ADHD	Attention-deficit/hyperactivity disorder
AED	Antiepileptic drugs
ASD	Autism spectrum disorder
ASM	Antiseizure medications
CIs	Confidence intervals
CNVs	Copy number variants
DRE	Drug-resistant epilepsy
EEG	Electroencephalography
GDD	Global developmental delay
GLUT1-DS	Glucose Transporter Type 1 Deficiency Syndrome
HSV2	Herpes Simplex Virus Type 2
ICD-10	International Classification of Diseases 10th Revision
ID	Intellectual disability
ILAE	International League Against Epilepsy
MRI	Magnetic resonance imaging
NGS	Next-generation sequencing
VUS	Variants of uncertain significance
WES	Whole-exome sequencing
WGS	Whole-genome sequencing
