# Supplementary material for: Nationwide Study of Pediatric Drug-Resistant Epilepsy in Estonia: Lower Incidence and Insights into Etiology
Source: Pediatr Rep. 2026 Jan 6;18(1):8. doi: 10.3390/pediatric18010008 (PMC12821569; doi:10.3390/pediatric18010008)
Supplement: Supplementary file 1 [file pediatrrep-18-00008-s001.zip › pediatrrep-3981509-supplementary.pdf]

Pathogenic sequence variants

Pt.	Gene	Transcript	c.	p.	variant type	Automated ACMG classification (Varsome)		Manually revised ACMG classification	CADD	REVEL	Zygosity	HGVS	Assay	Comments
1	*CDKL5*	NM_003159.3	c.2225_2228del	p.(Glu742Alafs*41)	frameshift	P	10P-0B	P	-	-	hemi (*de novo*)	NM_003159.3(CDKL5):c.2225_2228del p.(Glu742Alafs*41)	Panel	DOI: 10.1055/s-0036-1586730
2	*COL4A1*	NM_001845.6	c.4022-2del	p.?	splice site	LP	9P-0B	P	-	-	het (mat)	NM_001845.6(COL4A1):c.4022-2del p.?	Panel	
3	*CPA6*	NM_020361.5	c.1199G>A	p.(Arg400His)	missense	VUS	5P-0B	P	27.6	0.618	het (*de novo*)	NM_020361.5(CPA6):c.1199G>A p.(Arg400His)	Panel	
4	*CSNK2A1*	NM_177559.3	c.224dup	p.(Lys76Glufs*4)	frameshift	LP	9P-0B	P	-	-	het *(de novo)*	NM_177559.3(CSNK2A1):c.224dup p.(Lys76Glufs*4)	Exome	
5	*DNM1*	NM_004408.4	c.1197-8G>A	p.?	non coding	LB	1P-2B	P		-	het *(de novo)*	NM_004408.4(DNM1):c.1197-8G>A p.?	Exome	affects splicing
6	*DYNC1H1*	NM_001376.5	c.5756T>C	p.(Leu1919Pro)	missense	LP	6P-0B	P	28.5	0.858	het *(de novo)*	NM_001376.5(DYNC1H1):c.5756T>C p.(Leu1919Pro)	Panel	
7	*GABRG2*	NM_198904.4	c.964G>A	p.Ala322Thr	missense	VUS	4P-0B	P	25.7	0.647	het *(de novo)*	NM_198904.4(GABRG2):c.964G>A p.(Ala322Thr)	Panel	
8	*IRF2BPL*	NM_024496.4	c.240_243del	p.(Val81Profs*70)	frameshift	P	10P-0B	P	-	-	het *(de novo)*	NM_024496.4(IRF2BPL):c.240_243del p.(Val81Profs*70)	Genome	
9	*KCNQ2*	NM_172107.4	c.1762A>C	p.(Arg588=)	synonymous	VUS	5P-0B	P	22	-	het *(de novo)*	NM_172107.4(KCNQ2):c.1762A>C p.(Arg588=)	Exome	likely affects splicing
10	*KMT2D*	NM_003482.4	c.2579del	p.(Leu860Argfs*70)	frameshift	P	11P-0B	P	-	-	het *(de novo)*	NM_003482.4(KMT2D):c.2579del p.(Leu860Argfs*70)	Single gene testing	
11	*LAMB1*	NM_002291.3	c.4188+1G>C	p.?	non coding	P	13P-0B	P	34	-	c.het (mat)	NM_002291.3(LAMB1):c.4188+1G>C	Genome	

			c.2315-28A>G	p.?	non coding	LB	1P-4B	P	0.2	-	c.het (pat)	NM_002291.3(LAMB1):c.2315-28A>G	Genome	RNAseq confirms splicing aberration. Full classification in ClinVar: SCV003922126.1
12	*MECP2*	NM_004992.4	c.473C>T	p.(Thr158Met)	missense	P	23P-0B	P	26.3	0.945	het	NM_004992.4(MECP2):c.473C>T p.(Thr158Met)	Single gene testing	
13	*MECP2*	NM_001110792.2	c.509C>T	p.(Thr170Met)	missense	P	23P-0B	P	26.3	0.945	het	NM_001110792.2(MECP2):c.509C>T p.(Thr170Met)	Single gene testing	
14	*PCDH19*	NM_001184880.2	c.2057del	p.(Gly686Alafs*9)	frameshift	LP	9P-0B	P	-	-	het (*de novo*)	NM_001184880.2(PCDH19):c.2057del (p.Gly686Alafs*9)	Panel	
15	*PCDH19*	NM_020766.3	c.1091del	p.(Pro364Argfs*4)	frameshift	P	13P-0B	P	-	-	het (*de novo*)	NM_020766.3(PCDH19):c.1091del p.(Pro364Argfs*4)	Panel	
16	*PPT1*	NM_000310.4	c.364A>T	p.(Arg122Trp)	missense	P	13P-1B	P	27.4	-	hom	NM_000310.4(PPT1):c.364A>T p.(Arg122Trp)	Panel	both parents are carriers, autosomal recessive
17	*PRRT2*	NM_145239.3	c.649dup	p.(Arg217Profs*8)	frameshift	P	17P-0B	P	-	-	het	NM_145239.3(PRRT2):c.649dup p.(Arg217Profs*8)	Panel	
18	*SCN1A*	NM_001165963.4	c.2651G>A	p.(Gly884Asp)	missense	LP	7P-0B	P	25.7	0.988	het (*de novo*)	NM_001165963.4(SCN1A):c.2651G>A p.(Gly884Asp)	Panel	
19	*SCN1A*	NM_001165963.4	c.2837G>A	p.(Arg946His)	missense	P	21P-0B	P	28.1	0.987	het (*de novo*)	NM_001165963.4(SCN1A):c.2837G>A p.(Arg946His)	Panel	
20	*SLC2A1*	NM_006516.4	c.274C>T	p.(Arg92Trp)	missense	P	14P-0B	P	27.1	0.918	het	NM_006516.4(SLC2A1):c.274C>T p.(Arg92Trp)	Panel	likely de novo, not inherited from mother, father unavailable for testing
21	*SMARCB1*	NM_003073.5	c.362+1G>A	p.?	splice site	P	13P-0B	P	35	-	het (*de novo*)	NM_003073.5(SMARCB1):c.362+1G>A p.?	Panel	
22	*SYNGAP1*	NM_006772.3	c.3253del	p.(Arg1085Glyfs*45)	frameshift	LP	9P-0B	P	-	-	het (*de novo*)	NM_006772.3(SYNGAP1):c.3253del p.(Arg1085Glyfs*45)	Exome	

			c.3255del	p.(Pro1086Hisfs*44)	frameshift	LP	9P-0B	P	-	-	het (*de novo*)	NM_006772.3(SYNGAP1):c.3255del p.(Pro1086Hisfs*44)	Exome	
23	*TSC2*	NM_000548.5	c.1362-?_1716+?del (ex14-ex16del)					P			het (*de novo*)	NM_000548.5(TSC2):c.1362-?_1716+?del (ex14-ex16del)	Single gene testing	
24	*TSC2*	NM_000548.5	c.3203C>T	p.(Thr1068Ile)	missense	P	17P-0B	P	24.4	0.932	het (*de novo*)	NM_000548.5(TSC2):c.3203C>T p.(Thr1068Ile)	Panel	
25	*UNC13D*	NM_199242.3	c.2216_2239del	p.(Asn739_Gln746del)	in frame	VUS	3P-0B	P	-	-	c.het (pat)	NM_199242.3(UNC13D):c.2216_2239del p.(Asn739_Gln746del)	Unknown	considering phenotypic similarity and that the variant is in trans with the pathogenic frameshift variant, we categorise this as pathogenic
		NM_199242.3	c.3037dup	p.(Asp1013Glyfs*12)	frameshift	LP	9P-0B	P	-	-	c.het (mat)	NM_199242.3(UNC13D):c.3037dup p.(Asp1013Glyfs*12)	Unknown	
**Variants with unknown significance**														
26	*ALG13*	NM_001257237.2	c.1641A>T	p.(Gln547His)	missense	LB	1P-3B	VUS	16.3	0.07	hemi (mat)	NM_001257237.2(ALG13):c.1641A>T p.(Gln547His)	Panel	DOI: 10.1159/000448369
27	*CACNA1E*	NM_000721.4	c.457G>A	p.(Gly153Ser)	missense	VUS	3P-1B	VUS	30	0.861	het (pat)	NM_000721.4(CACNA1E): c.457G>A p.(Gly153Ser)	Panel	
28	*HCN1*	NM_021072.4	c.2375C>T	p.(Ser792Leu)	missense	VUS	1P-1B	VUS	24.9	0.291	het (mat)	NM_021072.4(HCN1):c.2375C>T p.(Ser792Leu)	Panel	maternally inherited, however mother also had childhood epilepsy
29	*KIF1A*	NM_004321.8	c.950A>G	p.(Glu317Gly)	missense	LP	7P-0B	VUS	31	0.912	het (pat)	NM_004321.8(KIF1A):c.950A>G p.(Glu317Gly)	Panel	
30	*RYR2*	NM_001035.3	c.836C>T	p.(Thr279Met)	missense	VUS	2P-0B	VUS	24.8	0.524	het	NM_001035.3(RYR2):c.836C>T p.(Thr279Met)	Exome	not inherited from mother, father unavailable for testing

31	*SCN2A*	NM_021007.3	c.5174A>G	p.(Asn1725Ser)	missense	VUS	3P-0B	VUS	24.2	0.635	het	NM_021007.3(SCN2A):c.5174A>G p.(Asn1725Ser)	Panel	
32	*SCN2A*	NM_021007.3	c.1112G>A	p.(Ser371Asn)	missense	VUS	4P-0B	VUS	26.3	0.742	het (mat)	NM_021007.3(SCN2A):c.1112G>A p.(Ser371Asn)	Panel	
33	*SLC9A6*	NM_006359.3	c.472C>T	p.(Pro158Ser)	missense	VUS	3P-1B	VUS	24.4	0.648	hemi (mat)	NM_006359.3(SLC9A6):c.472C>T p.(Pro158Ser)	Panel	brother without epilepsy has the same variant
34	*SPTAN1*	NM_001130438.3	c.6422T>G	p.(Phe2141Cys)	missense	VUS	5P-1B	VUS	32	0.93	het	NM_001130438.3(SPTAN1):c.6422T>G p.(Phe2141Cys)	Panel	not inherited from mother, father unavailable for testing
35	*WNK3*	NM_020922.5	c.4996C>T	p.(Leu1666Phe)	missense	LB	1P-3B	VUS	24.2	0.053	hemi (mat)	NM_020922.5(WNK3):c.4996C>T p.(Leu1666Phe)	Exome	
**Novel disease gene candidates**														
36	*ACSL5*	NM_016234.4	c.1853A>G	p.(Tyr618Cys)	missense	VUS	5P-0B	GUS	27.3	0.694	c.het (pat)	NM_016234.4(ACSL5):c.1853A>G p.(Tyr618Cys)	Genome	
			c.*760A>G	p.?	non coding, 3'UTR	LB	1P-4B	GUS	9.9	-	c.het (mat)	NM_016234.4(ACSL5):c.*760A>G p.?	Genome	
	RNU2-2P	ENST00000410396.1	n.35A>G		non coding			P	10.9	-	het (*de novo*)	ENST00000410396.1(RNU2-2P):n.35A>G	Genome	https://www.medrxiv.org/content/10.1101/2024.09.03.24312863v1.full-text
37	*DSCAM*	NM_001389.5	c.5116G>A	p.(Val1706Ile)	missense	VUS	2P-1B	GUS	23.3	0.115	het (*de novo*)	NM_001389.5(DSCAM):c.5116G>A p.(Val1706Ile)	Exome	
	LMTK3	NM_001388485.1	c.2190dup	p.(Glu731Argfs*809)	frameshift	VUS	4P-4B	GUS	-	-	het (*de novo*)	NM_001388485.1(LMTK3):c.2190dup p.(Glu731Argfs*809)	Exome	
38	*SIRT6*	NM_016539.4	c.701G>A	p.(Gly234Asp)	missense	VUS	1P-0B	GUS	26.1	-	c.het (pat)	NM_016539.4(SIRT6):c.701G>A p.(Gly234Asp)	Genome	patient 38 and 39 are siblings (triplets). Functional studies in process

			c.615-8G>A	p.?	non coding	VUS	5P-0B	GUS	20.9	-	c.het (mat)	NM_016539.4(SIRT6):c.615-8G>A p.?	Genome	splice-site, functional studies in process
39	*SIRT6*	NM_016539.4	c.701G>A	p.(Gly234Asp)	missense	VUS	1P-0B	GUS	26.1	-	c.het (pat)	NM_016539.4(SIRT6):c.701G>A p.(Gly234Asp)	Genome	patient 38 and 39 are siblings (triplets). Functional studies in process
			c.615-8G>A	p.?	non coding	VUS	5P-0B	GUS	20.9	-	c.het (mat)	NM_016539.4(SIRT6):c.615-8G>A p.?	Genome	splice-site, functional studies in process

Abbreviations: Pt.- patient, B - benign, LB - likely benign, VUS - variant of uncertain significance, LP - likely pathogenic, P - pathogenic, GUS - gene of uncertain significance, het - heterozygous, c.het - compound heterozygous, hom - homozygous, hemi - hemizygous, mat - maternal, pat - paternal

Chromosomal aberrations

Patient	ISCN	Automated ACMG classification (CNV-ClinViewer)	Manually revised ACMG classification	Assay	Comments
40	arr[hg19] 15q13.3(30955149_32515681)x1	P	P/risk factor	chromosomal microarray	
41	arr[hg19] 22q11.21(18656495_19008108)x3 (mat)	VUS	P/risk factor	chromosomal microarray	maternal
42	arr[hg19] 10q26.13q26.3(127371995_135430043)x1	P	P	chromosomal microarray	
43	arr[hg19] 22q11.21(18877787_21462353)x1	P	P	chromosomal microarray	
44	46,XY,r(14)(p12q32)	-	P	karyotype	*de novo*
